# Cellular Basis of Bitter-Driven Aversive Behaviors in *Drosophila* Larva

**DOI:** 10.1523/ENEURO.0510-19.2020

**Published:** 2020-04-08

**Authors:** Jaekyun Choi, Seungyun Yu, Min Sung Choi, Sooin Jang, I Joon Han, G. Larisa Maier, Simon G. Sprecher, Jae Young Kwon

**Affiliations:** 1 Department of Biological Sciences, Sungkyunkwan University, Suwon 16419, Republic of Korea; 2 School of Medicine, Sungkyunkwan University, Suwon 16419, Republic of Korea; 3 Department of Biology, Institute of Zoology, University of Fribourg, Fribourg CH-1700, Switzerland

**Keywords:** Drosophila, neural circuit, taste

## Abstract

Feeding, a critical behavior for survival, consists of a complex series of behavioral steps. In *Drosophila* larvae, the initial steps of feeding are food choice, during which the quality of a potential food source is judged, and ingestion, during which the selected food source is ingested into the digestive tract. It remains unclear whether these steps employ different mechanisms of neural perception. Here, we provide insight into the two initial steps of feeding in *Drosophila* larva. We find that substrate choice and ingestion are determined by independent circuits at the cellular level. First, we took 22 candidate bitter compounds and examined their influence on choice preference and ingestion behavior. Interestingly, certain bitter tastants caused different responses in choice and ingestion, suggesting distinct mechanisms of perception. We further provide evidence that certain gustatory receptor neurons (GRNs) in the external terminal organ (TO) are involved in determining choice preference, and a pair of larval pharyngeal GRNs is involved in mediating both avoidance and suppression of ingestion. Our results show that feeding behavior is coordinated by a multistep regulatory process employing relatively independent neural elements. These findings are consistent with a model in which distinct sensory pathways act as modulatory circuits controlling distinct subprograms during feeding.

## Significance Statement

Here, we provide molecular and cellular evidence that feeding can indeed be dissected into two distinct steps, namely the determination of preference and initiation of ingestion. We find that bitter tastants have individual characteristics when negatively affecting feeding, with most chemicals negatively affecting both preference and ingestion, and certain chemicals negatively affecting only preference. These characteristics are due to different sensory neurons being responsible for detecting different bitter compounds. The different sensory neurons appear to act in relatively independent neural circuits to differentially affect the multiple steps that comprise feeding.

## Introduction

The regulation of feeding is critical for the survival of animals. Feeding is comprised of a series of behavioral modules or subprograms that can be regulated at multiple levels (Pool and Scott, 2014). At the initiation step of a meal, the chemical composition of a food source provides important information regarding the palatability and quality of food, which in turn is essential for the decision of whether to feed on the respective food. Sweet taste is generally recognized as a preferred source of nutrients, while bitter taste is generally recognized as toxic material to be avoided. Animals can regulate feeding by sensing bitterness, subsequently avoiding the bitter food, and suppressing ingestion. *Drosophila* larvae have been used as an effective model to study the cellular and molecular bases of behavioral responses caused by bitterness. (El-Keredy et al., 2012; Apostolopoulou et al., 2014; König et al., 2014; Choi et al., 2016; Kim et al., 2016; van Giesen et al., 2016b).

The major chemosensory organs of *Drosophila* larvae are located in bilaterally symmetrical pairs on the head, and are composed of three external sense organs: the terminal organ (TO), ventral organ (VO), and dorsal organ (DO) as well as three sets of pharyngeal sensilla (Singh and Singh, 1984; Stocker, 1994; Python and Stocker, 2002; Gendre et al., 2004; Gerber and Stocker, 2007). Also, numerous studies have provided functional evidence that these organs are indeed involved in taste perception (Python and Stocker, 2002; Apostolopoulou et al., 2014, 2015; Choi et al., 2016; Kim et al., 2016; van Giesen et al., 2016b).

Members of the gustatory receptor (*Gr*), ionotropic receptor (*IR*), and pickpocket (*ppk*) gene families are involved in chemosensory perception, and are expressed in the larval gustatory neurons (Liu et al., 2003; Colomb et al., 2007; Thorne and Amrein, 2008; Kwon et al., 2011; Mishra et al., 2013; Stewart et al., 2015). The expression of some *Ir*, *Ppk*, and *Gr* genes have been mapped to neurons of individual sensilla in the TO (Rist and Thum, 2017), and expression of *Gr-GAL4* drivers has been mapped to individual GRNs in the TO and pharyngeal sense organs (Kwon et al., 2011; Choi et al., 2016). The Gr-to-neuron map of the TO and pharyngeal sense organs that was constructed based on *Gr-GAL4* driver expression shows that many GRNs in *Drosophila* larva express receptors that have been associated with bitter taste in the adult fly, suggesting the sensing of bitterness by these GRNs. However, co-expression of several receptors differ in the larva and the adult, and numerous Grs are stage specific. Accumulating evidence for the multimodality of taste sensing in the larva (van Giesen et al., 2016b) and complex receptor co-expression requirements for taste discrimination in the fly raise the question of how these neurons coordinate taste perception (Jaeger et al., 2018).

We here show that feeding initiation in *Drosophila* larvae can be divided into two distinct steps, choice and ingestion, with choice being the process of selecting a potential food source, and ingestion the process of moving food down the pharyngeal tract into the digestive system. We tested a wide panel of twenty-two putative bitter compounds and found that some tastants have different impacts on ingestion and choice, contrary to our initial assumption that tastants would generally cause similar responses in these two behavioral paradigms. Using molecular genetics and calcium imaging, we provide evidence that caffeine (CAF) is detected by a pair of neurons in the dorsal pharyngeal sensilla (DPS) to induce avoidance movement and reduction of ingestion, and denatonium is detected by neurons in the TO to induce only avoidance movement.

## Materials and Methods

### *Drosophila* stocks and transgenic flies

Flies were cultured on standard cornmeal agar medium at room temperature (23 ± 2°C). *wCS* was used a control for behavioral assays. All *Gr-GAL4* transgenic lines used in this study were previously described (Kwon et al., 2011). The following fly lines were used: *Gr33a^1^* (Moon et al., 2009), *UAS-Kir2.1* (Baines et al., 2001), *C7-GAL4* (van Giesen et al., 2016b), *UAS-Gr33a* (Moon et al., 2009), *UAS-Gr59c* (Weiss et al., 2011), and *UAS-GCaMP6m* (Chen et al., 2013).

### Tastants

The highest purity tastants commercially available were purchased for use in behavioral assays. Stock solutions of the tastants dissolved in water were mixed with 1% agarose solution (ingestion assay) or 1.5% agarose solution (choice assay) to prepare the plates used in the behavioral assays. The following 22 bitter compounds were selected to test in this study: atropine (ATR; A0132, Sigma), berberine chloride (BER; B3251, Sigma), CAF (27600, Sigma), (+)-catechin (CAT; ALX-385-017, Enzo), coumarin (COU; C4261, Sigma), *N,N*-diethyl-*m*-toluamide (DEET; PS-902, Supelco), denatonium benzoate (DEN; D5765, Aldrich), escin (ESC; E1378, Sigma), gallic acid (GAA; G7384, Sigma), gibberellic acid (GIA; 63492, Aldrich), harmaline (HAR; 51330, Aldrich), (-)-lobeline hydrochloride (LOB; 141879, Aldrich), (-)-nicotine (NIC; 36733, Fluka), *N*-phenylthiourea (PTU; P7629, Sigma), quinine hydrochloride dihydrate (QUI; 22630, Sigma), saponin (SAP; 102855, MP Bio), *D-*(+)-sucrose octaacetate (SOA; 84112, Fluka), strychnine nitrate (STR; S0093, TCI), tannic acid (TAA; 194859, MP Bio), theobromine (THB; T4500, Sigma), theophylline anhydrous (TPH; 103024, MP Bio), and umbelliferone (UMB; 93 979, Sigma).

### Behavioral assays

Third instar larvae were used for all behavioral experiments. All assays were conducted with 5 d-old larvae after egg laying. All larvae were thoroughly washed with distilled water. For the ingestion assay, we followed a previously described protocol (Choi et al., 2016). Briefly, 60-mm Petri dishes (SPL 10060) were filled with 1% agarose solution + 1% indigo carmine (Sigma, 57000; control plates), or 1% agarose solution + 1% indigo carmine + bitter substance (experimental plates). Thirty third instar larvae were placed in the center of the plate. After 90 min of feeding, larvae were collected and washed with distilled water. Next, larvae were homogenized using a pistil in 1 M 60-μl L-ascorbic acid (Sigma, A7506). After centrifugation at 13,200 rpm for 10 min, the blue supernatant was transferred to a 1-μm-pore FAPD column and centrifuged at 13,200 rpm for 3 min. The filtered supernatant was transferred to a 96-well plate (SPL 30096) and absorbance was measured at 630 nm with a spectrophotometer (BioTek EL800). The relative ingestion index (I.I.) was derived by calculating the difference in absorbance O.D. (optical density) between the control and experimental groups: I.I. = (experimental O.D. – empty O.D.) – (dye-only control O.D. – empty O.D.)/(dye-only control O.D. – empty O.D.). The dye only control O.D. was measured for every experiment, for every genotype, on the same day. An I.I. value of 0 indicates that larvae on the test plate ate as much as larvae on the dye-only control plate, while I.I. = −1 indicates that larvae on the test plate did not eat at all, and I.I. > 0 indicates that larvae on the test plate ate more than larvae on the dye-only control plate.

For the choice preference assay, we used a previously described method with modifications (Kim et al., 2016). Ninety-millimeter Petri dishes (SPL 10096) were first filled with 1.5% agarose solution and allowed to cool. After dividing the cooled agarose into quadrants and carefully removing the second and fourth agarose quadrants, the now empty quadrants were filled with 1.5% agarose containing a bitter substance. After the plate fully solidified, 20 third instar larvae were placed in the center of the prepared quadrant plate. The number of larvae on each quadrant were counted after 8 min, with the first and third quadrant being the agarose only control and the second and fourth quadrant containing the bitter substance. The choice preference index (P.I.) was calculated as follows: P.I. = [N_bitter tastant_ – N_agarose only_]/[N_total_]. A negative P.I. value indicates that the larvae showed aversive behavior toward the tested putative bitter substance.

For mouth-hook contraction, body wall contraction, and bending behavior assay, we used a previously described method with some modifications (Shen, 2012; Bhatt and Neckameyer, 2013). 90-mm Petri dishes (SPL 10096) were filled with 1% agarose (control plate), or 1% agarose with bitter substance (experimental plate). One larva was placed on the center of the plate, and video recording was done for 60 s after a 90-s acclimation. We analyzed the video files by counting the number of each mouth-hook contraction, body wall contraction, and bending behavior. Mouth-hook contraction was assessed as pharyngeal muscle movement, body wall contraction is locomotion movement, and bending was assessed as when the larval body was bent at an angle >45°. Each behavior was compared between larvae on 1% agarose plate (control), or 1% agarose plate with bitter substance (experiment).

### GCaMP imaging

To record calcium responses, early stage third instar larvae were dissected in modified AHL, adult hemolymph-like-saline (108 mM NaCl, 5 mM KCl, 8.2 mM MgCl_2_, 4 mM NaHCO_3_, 1 mM NaH_2_PO_4_, and 5 mM HEPES, pH 7.5 in Millipore water). To enable recording in DP1 and DP2, the cuticle surrounding the tip of the head was removed and the head was introduced into the microfluidic chip chamber to expose chemosensory organs to the liquid passing through the channel (van Giesen et al., 2016a). A drop of 2% agarose diluted in AHL saline was used to close the channel. Measurements were conducted as follows: a 10-s period of washing (Millipore water) followed by a 20-s period of stimulation and another 10 s of washing. Changes in fluorescence were calculated as follows:

$$\Delta \text{F}/\text{F}\;\left(\%\right)=\left({\text{F}}_{\text{peak}}-{\;\text{F}}_{0}\right)*\;\text{1}00/{\;\text{F}}_{0}.$$

F_0_ was calculated from five frames during the prestimulation phase of the first 100-frame time period. F_peak_ was taken as the point of highest intensity measured during the time of stimulation. A Zeiss LSM 700 confocal microscope was used for imaging. For the analysis of calcium imaging measurements, ImageJ was used and changes in fluorescence were calculated in Microsoft Excel.

### Experimental design and statistical analyses

Statistical analyses were conducted using GraphPad Prism 5 and IBM SPSS Statistics 20. Behavior data are mostly presented as a box plot, with the middle line representing the median, + the mean, and the box boundaries and whiskers representing 25%/75% and 10%/90%, respectively. For Figures 2, 3*A*, 4*A*, 5*A*, 6*A*, normality was tested. Data were considered to be normally distributed if it passed either the Kolmogorov–Smirnov test or the Shapiro–Wilk normality test. For Figure 2, one-way ANOVA followed by Dunnett’s multiple comparison test was used. For Figures 3*A*, 4*A*, 6*A*, one-way ANOVA followed by the Newman–Keuls method was performed if the data were normally distributed. If the data were not normally distributed, the Kruskal–Wallis test followed by Dunn’s multiple comparison test was performed. For Figure 5*A*, one-way ANOVA was followed by uncorrected Fisher’s least significant difference (LSD) test. For Figures 3*B*,*C*, 4*B*,*C*, 5*B*,*C*, 6*B*, two-way ANOVA followed by the Bonferroni *post hoc* test was performed. The Mann–Whitney *U* test was used for pair-wise comparison of GCaMP imaging data. Asterisks shown in figures signify statistical significance (*p <* *0.05, **p <* *0.01, ***p <* *0.001). Additional details are described in the figure legends and Table 1.

**Table 1:** Statistical analysis summary

Figure		Test comparison	Statistical test	Result	*Post hoc* test	**post-hoc comparison**	**Adjusted P value**
2*A*	Mouth-hook contraction	Comparisons between tastants	One-way ANOVA	*F*_(3,102)_ = 7.901, *P* < 0.0001	Dunnett's multiple comparison test	Control vs CAF	0.001
						Control vs DEN	>0.999
						Control vs NIC	<0.001
	Bending			*F*_(3,102)_ = 8.846, *P* < 0.0001		Control vs CAF	0.092
						Control vs DEN	0.026
						Control vs NIC	0.026
	Body wall contraction			*F*_(3,102)_ = 16.94, *P* < 0.0001		Control vs CAF	0.598
						Control vs DEN	0.628
						Control vs NIC	<0.001
2*B*	Mouth-hook contraction	Comparisons between tastants	One-way ANOVA	*F*_(3,76)_ = 4.355, *P* = 0.0069	Dunnett's multiple comparison test	Control vs CAF	0.473
						Control vs DEN	0.060
						Control vs NIC	0.995
	Bending			*F*_(3,76)_ = 4.773, *P* = 0.0042		Control vs CAF	0.312
						Control vs DEN	0.923
						Control vs NIC	0.078
	Body wall contraction			*F*_(3,76)_ = 11.90, *P* < 0.0001		Control vs CAF	>0.999
						Control vs DEN	0.765
						Control vs NIC	<0.001
2*C*	Mouth-hook contraction	Comparisons between tastants	One-way ANOVA	*F*_(2,56)_ = 0.2104, *P* = 0.8109	Dunnett's multiple comparison test	Control vs SUC	>0.999
						Control vs FRU	0.793
	Bending		Kruskal–Wallis test	0.9784, *P* = 0.6131	Dunn's multiple comparison test	Control vs SUC	>0.05
						Control vs FRU	>0.05
	Body wall contraction		One-way ANOVA	*F*_(2,56)_ = 0.1099, *P* = 0.8961	Dunnett's multiple comparison test	Control vs SUC	0.990
						Control vs FRU	0.863
3*A*		Comparisons between genotypes	Kruskal–Wallis test	39.44, *P* < 0.0001	Dunn's multiple comparison test	*UAS-Kir2.1/+* vs *Gr33a>Kir2.1*	<0.05
						*Gr33a-GAL4/+* vs *Gr33a>Kir2.1*	<0.05
						*UAS-Kir2.1/+* vs *Gr39b>Kir2.1*	<0.05
						*Gr39b-GAL4/+* vs *Gr39b>Kir2.1*	<0.01
						*UAS-Kir2.1/+* vs *Gr23a>Kir2.1*	>0.05
						*Gr23a-GAL4/+* vs *Gr23a>Kir2.1*	>0.05
						*UAS-Kir2.1/+* vs *Gr22a>Kir2.1*	>0.05
						*Gr22a-GAL4/+* vs *Gr22a>Kir2.1*	>0.05
						*UAS-Kir2.1/+* vs *Gr36b>Kir2.1*	>0.05
						*Gr36b-GAL4/+* vs *Gr36b>Kir2.1*	>0.05
3*B*		Interaction between genotype and chemical	Two-way ANOVA	*F*_(9,356)_ = 4.149, *P* < 0.0001	Bonferroni's multiple comparison test	Control vs 100 mM CAF (*Gr33a-GAL4/+*)	<0.05
						Control vs 100 mM CAF (*Gr33a>Kir2.1*)	<0.05
						Control vs 100 mM CAF (*Gr39b-GAL4/+*)	<0.05
						Control vs 100 mM CAF (*Gr39b>kir2.1*)	>0.05
						Control vs 100 mM CAF (*Gr23a-GAL4/+*)	<0.01
						Control vs 100 mM CAF (*Gr23a>kir2.1*)	<0.01
						Control vs 100 mM CAF (*Gr22a-GAL4/+*)	>0.05
						Control vs 100 mM CAF (*Gr22a>kir2.1*)	>0.05
						Control vs 100 mM CAF (*Gr36b-GAL4/+*)	<0.01
						Control vs 100 mM CAF (*Gr36b>kir2.1*)	>0.05
3*C*		Interaction between genotype and chemical	Two-way ANOVA	*F*_(9,356)_ = 1.837, *P* = 0.0606	Bonferroni's multiple comparison test	Control vs 100 mM CAF (*Gr33a-GAL4/+*)	>0.05
						Control vs 100 mM CAF (*Gr33a>Kir2.1*)	>0.05
						Control vs 100 mM CAF (*Gr39b-GAL4/+*)	>0.05
						Control vs 100 mM CAF (*Gr39b>kir2.1*)	>0.05
						Control vs 100 mM CAF (*Gr23a-GAL4/+*)	<0.01
						Control vs 100 mM CAF (*Gr23a>kir2.1*)	<0.05
						Control vs 100 mM CAF (*Gr22a-GAL4/+*)	>0.05
						Control vs 100 mM CAF (*Gr22a>kir2.1*)	>0.05
						Control vs 100 mM CAF (*Gr36b-GAL4/+*)	>0.05
						Control vs 100 mM CAF (*Gr36b>kir2.1*)	>0.05
4*A*		Comparisons between genotypes	One-way ANOVA	*F*_(10,64)_ = 15.38, *P* < 0.0001	Newman–Keuls multiple comparison test	*UAS-Gr33a/+* vs *Gr33a>Gr33a*	<0.001
						*Gr33a-GAL4/+* vs *Gr33a>Gr33a*	<0.001
						*UAS-Gr33a/+* vs *Gr39b>Gr33a*	<0.001
						*Gr39b-GAL4/+* vs *Gr39b>Gr33a*	<0.001
						*UAS-Gr33a/+* vs *Gr22d>Gr33a*	<0.001
						*Gr22d-GAL4/+* vs *Gr22d>Gr33a*	<0.001
						*UAS-Gr33a/+* vs *Gr22a>Gr33a*	>0.05
						*Gr22a-GAL4/+* vs *Gr22a>Gr33a*	>0.05
						*UAS-Gr33a/+* vs *Gr36b>Gr33a*	>0.05
						*Gr36b-GAL4/+* vs *Gr36b>Gr33a*	>0.05
4*B*		Interaction between genotype and chemical	Two-way ANOVA	*F*_(9,402)_ = 2.597, *P* = 0.0064	Bonferroni's multiple comparison test	Control vs 100 mM CAF (*Gr33a-GAL4/+*)	>0.05
						Control vs 100 mM CAF (*Gr33a>Gr33a*)	<0.05
						Control vs 100 mM CAF (*Gr39b-GAL4/+*)	>0.05
						Control vs 100 mM CAF (*Gr39b>Gr33a*)	>0.05
						Control vs 100 mM CAF (*Gr22d-GAL4/+*)	>0.05
						Control vs 100 mM CAF (*Gr22d>Gr33a*)	>0.05
						Control vs 100 mM CAF (*Gr22a-GAL4/+*)	>0.05
						Control vs 100 mM CAF (*Gr22a>Gr33a*)	>0.05
						Control vs 100 mM CAF (*Gr36b-GAL4/+*)	>0.05
						Control vs 100 mM CAF (*Gr36b>Gr33a*)	>0.05
4*C*		Interaction between genotype and chemical	Two-way ANOVA	*F*_(9,402)_ = 2.084, *P* = 0.0299	Bonferroni's multiple comparison test	Control vs 100 mM CAF (*Gr33a-GAL4/+*)	>0.05
						Control vs 100 mM CAF (*Gr33a>Gr33a*)	>0.05
						Control vs 100 mM CAF (*Gr39b-GAL4/+*)	>0.05
						Control vs 100 mM CAF (*Gr39b>Gr33a*)	<0.05
						Control vs 100 mM CAF (*Gr22d-GAL4/+*)	>0.05
						Control vs 100 mM CAF (*Gr22d>Gr33a*)	>0.05
						Control vs 100 mM CAF (*Gr22a-GAL4/+*)	>0.05
						Control vs 100 mM CAF (*Gr22a>Gr33a*)	>0.05
						Control vs 100 mM CAF (*Gr36b-GAL4/+*)	>0.05
						Control vs 100 mM CAF (*Gr36b>Gr33a*)	>0.05
5*A*		Comparisons between genotypes	One-way ANOVA	*F*_(13,106)_ = 7.609, *P* < 0.0001	Uncorrected Fisher's LSD test	*C7-GAL4/+* vs *C7>Kir2.1*	0.032
						*Gr22a-GAL4/+* vs *Gr22a>Kir2.1*	0.002
						*C7,Gr22a-GAL4/+* vs *C7,Gr22a>kir2.1*	0.001
						*Gr28b.e-GAL4/+* vs *Gr28b.e>Kir2.1*	0.005
						*C7,Gr28b.e-GAL4/+* vs *C7,Gr28b.e>kir2.1*	<0.001
						*Gr59c-GAL4/+* vs *Gr59c>Kir2.1*	0.368
						*C7,Gr59c-GAL4/+* vs *C7,Gr59c>kir2.1*	<0.001
						*C7>Kir2.1* vs *C7,Gr22a>Kir2.1*	0.019
						*Gr22a>Kir2.1* vs *C7,Gr22a>Kir2.1*	0.002
						*C7>Kir2.1* vs *C7,Gr28b.e>Kir2.1*	0.044
						*Gr28b.e>Kir2.1* vs *C7,Gr28b.e>Kir2.1*	0.012
						*C7>Kir2.1* vs *C7,Gr59c>Kir2.1*	0.245
						*Gr59c>Kir2.1* vs *C7,Gr59c>Kir2.1*	0.018
5*B*		Interaction between genotype and chemical	Two-way ANOVA	*F*_(13,521)_ = 0.7760, *P* = 0.6860	Bonferroni's multiple comparison test	Control vs 100 mM CAF (*C7-GAL4/+*)	>0.05
						Control vs 100 mM CAF (*C7>Kir2.1*)	>0.05
						Control vs 100 mM CAF (*Gr22a-GAL4/+*)	>0.05
						Control vs 100 mM CAF (*Gr22a>Kir2.1*)	>0.05
						Control vs 100 mM CAF (*Gr22a,C7-GAL4/+*)	>0.05
						Control vs 100 mM CAF (*Gr22a,C7>Kir2.1*)	>0.05
						Control vs 100 mM CAF (*Gr28b.e-GAL4/+*)	>0.05
						Control vs 100 mM CAF (*Gr28b.e>Kir2.1*)	>0.05
						Control vs 100 mM CAF (*Gr28b.e,C7-GAL4/+*)	>0.05
						Control vs 100 mM CAF (*Gr28b.e,C7>Kir2.1*)	>0.05
						Control vs 100 mM CAF (*Gr59c-GAL4/+*)	>0.05
						Control vs 100 mM CAF (*Gr59c>Kir2.1*)	>0.05
						Control vs 100 mM CAF (*Gr59c,C7-GAL4/+*)	>0.05
						Control vs 100 mM CAF (*Gr59c,C7>Kir2.1*)	>0.05
5*C*		Interaction between genotype and chemical	Two-way ANOVA	*F*_(13,521)_ = 1.619, *P* = 0.0761	Bonferroni's multiple comparison test	Control vs 100 mM CAF (*C7-GAL4/+*)	>0.05
						Control vs 100 mM CAF (*C7>Kir2.1*)	>0.05
						Control vs 100 mM CAF (*Gr22a-GAL4/+*)	>0.05
						Control vs 100 mM CAF (*Gr22a>Kir2.1*)	>0.05
						Control vs 100 mM CAF (*Gr22a,C7-GAL4/+*)	>0.05
						Control vs 100 mM CAF (*Gr22a,C7>Kir2.1*)	>0.05
						Control vs 100 mM CAF (*Gr28b.e-GAL4/+*)	<0.05
						Control vs 100 mM CAF (*Gr28b.e>Kir2.1*)	>0.05
						Control vs 100 mM CAF (*Gr28b.e,C7-GAL4/+*)	>0.05
						Control vs 100 mM CAF (*Gr28b.e,C7>Kir2.1*)	>0.05
						Control vs 100 mM CAF (*Gr59c-GAL4/+*)	>0.05
						Control vs 100 mM CAF (*Gr59c>Kir2.1*)	>0.05
						Control vs 100 mM CAF (*Gr59c,C7-GAL4/+*)	<0.05
						Control vs 100 mM CAF (*Gr59c,C7>Kir2.1*)	>0.05
5*F*		Comparisons between tastants	Mann–Whitney *U* test			Water vs 10 mM denatonium (*Gr59c>GCaMP6m*)	0.009
						Water vs 10 mM denatonium (*C7>GCaMP6m*)	0.013
6*A*		Comparisons between genotypes	One-way ANOVA	*F*_(4,35)_ = 10.73, *P* < 0.0001	Newman–Keuls multiple comparison test	*UAS-Gr59c/+* vs *Gr22d>Gr59c*	<0.01
						*Gr22d-GAL4* vs *Gr22d>Gr59c*	<0.01
						*UAS-Gr59c/+* vs *Gr39b>Gr59c*	<0.001
						*Gr39b-GAL4* vs *Gr39b>Gr59c*	<0.01
6*B*		Interaction between genotype and chemical	Two-way ANOVA	*F*_(4,182)_ = 6.100, *P* = 0.0001	Bonferroni's multiple comparison test	Control vs 10 mM denatonium (*UAS-Gr59c/+*)	>0.05
						Control vs 10 mM denatonium (*Gr22d-GAL4/+*)	>0.05
						Control vs 10 mM denatonium (*Gr22d>Gr59c*)	<0.001
						Control vs 10 mM denatonium (*Gr39b-GAL4/+*)	>0.05
						Control vs 10 mM denatonium (*Gr39b>Gr59c*)	<0.001
6*E*		Comparisons between tastants	Mann–Whitney *U* test			Water vs 10 mM denatonium (*Gr39b>GCaMP6m*)	>0.05
						Water vs 10 mM denatonium (*Gr39b>Gr59c,GCaMP6m*)	<0.01

## Results

### Individual putative bitter tastants elicit distinct ingestion and choice preference behavior responses

**Figure 1.**
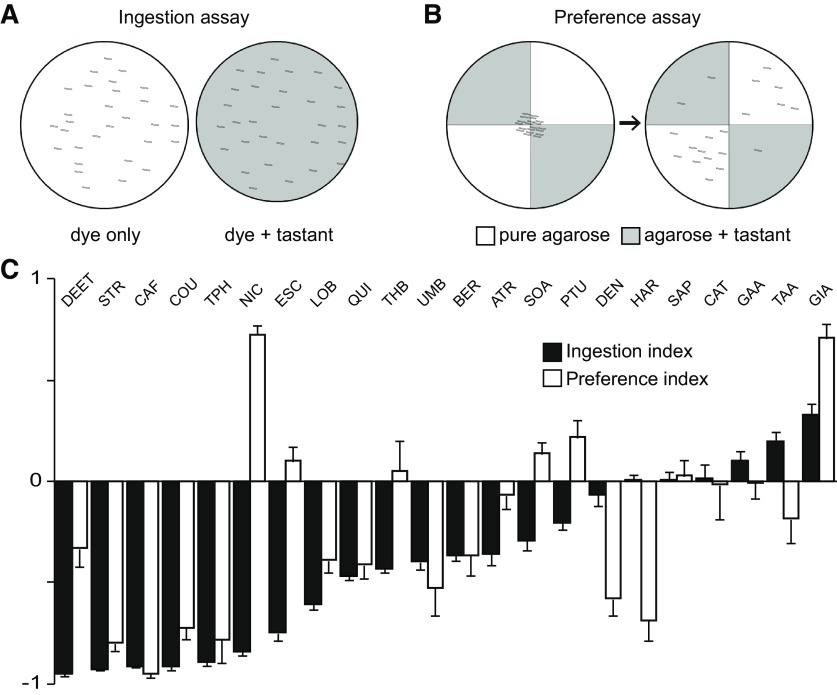
Responses toward putative bitter tastants in larval ingestion and choice assays. ***A***, Schematic drawing of the ingestion assay. Two agarose gel plates were used, with one containing only 1% indigo carmine dye (left, white) and one also containing a bitter substance (right, shaded). Thirty larvae were placed on each plate and allowed to forage and feed freely for 90 min. The ratio of dye ingested by the two groups was measured to calculate the I.I.. ***B***, For the choice preference assay, larvae were placed onto a quadrant plate made with two types of agarose gel: agarose only (white quadrants) and tastant included (shaded quadrants). After 8 min, the number of larvae on each quadrant was counted to analyze the larval behavioral response to the compound. The short lines in ***A***, ***B*** indicate larvae. ***C***, Comparison of ingestion and choice preference behavioral responses for the twenty-two bitter compounds tested. Compounds are arranged in order of increasing I.I. values from left to right. Each data point was derived from 6 < *n*. Error bars are SEM. The following concentrations were used: 10 mM ATR, 5 mM BER, 100 mM CAF, 10 mM CAT, 10 mM COU, 1% DEET, 10 mM DEN, 1 mM ESC, 50 mM GAA, 10 mM GIA, 5 mM HAR, 10 mM LOB, 10 mM NIC, 1 mM PTU, 10 mM QUI, 0.1% SAP, 1 mM SOA, 100 mM STR, 1 mM TAA, 30 mM THB, 100 mM TPH, and 10 mM UMB (Extended Data Figure 1-1).

In two previous studies, we measured larval preference (Kim et al., 2016) and ingestion behavior (Choi et al., 2016) toward putative bitter chemicals that were expected to elicit an aversive response. Interestingly, certain chemicals elicited differential responses between the two paradigms. As an example, denatonium caused larval avoidance in a dose-dependent manner (Kim et al., 2016), but did not affect ingestion even at the highest tested concentration of 10 mM (Choi et al., 2016). Here, we re-analyzed and quantitatively measured the two larval behaviors elicited by the bitter chemicals: ingestion (Fig. 1*A*) and choice behavior for substrate preference (Fig. 1*B*). We allowed larvae to feed for 90 min on control plates with agarose and dye or experimental plates with added tastants before quantifying and comparing the amounts ingested (Fig. 1*C*; for details, see Materials and Methods). An I.I. of 0 indicates that experimental larvae ingested the same amount as control larvae fed on dye only-agarose, and an I.I. of −1 indicates that experimental larvae did not ingest at all. An I.I. larger than 0 indicates that the tastant is a positive effector of ingestion, while an I.I. between 0 and −1 indicates that the tastant is a negative effector. In choice preference behavior assays, larvae were placed in the center of plates containing two quadrants with plain agarose and two quadrants with agarose and bitter tastants (Fig. 1*B*). We excluded the possible influence of sugar (for details, see Materials and Methods), in contrast to the previous study where sugar only and sugar plus putative bitter chemical were compared (Kim et al., 2016). The number of larvae on each quadrant was counted after 8 min to calculate the choice P.I. P.I. values between 0 and 1 indicate attraction, while values between 0 and −1 indicate aversion.

10.1523/ENEURO.0510-19.2020.f1-1 Extended Data Figure 1-1 The toxic effects of nicotine that block larval movement. As marked in the left uppermost panel, two quadrants of the plate are the agarose-only control, and the remaining two quadrants contain agarose with nicotine. Twenty larvae were placed in the center of the plate, and images of the plate were taken, starting from the upper left corner and moving to the lower right corner, at 40-s intervals. Therefore, the image of the upper left corner is at 0 s, and the image of the lower right corner at 7 min 20 s. The asterisks indicate larvae that have almost stopped moving. Download Figure 1-1, EPS file.

We found that CAF, STR, and TPH act as conventional bitter tastants that reduce ingestion and induce aversion (Fig. 1*C*); 100 mM CAF caused an I.I. of −0.911, with larvae barely eating agarose containing 100 mM CAF, and a P.I. of −0.948, with most larvae showing aversion toward CAF. Tastants such as DEET, COU, LOB, QUI, UMB, and BER show a similar tendency. Consistent with our previous observations, some tastants elicited differential effects on ingestion and preference behavior, which is counterintuitive to the general assumption that tastants would generally have similar effects on ingestion and preference. NIC and ESC cause a strong negative ingestion response but appear neutral or attractive in the choice preference assay (Fig. 1*C*). In contrast, DEN and HAR do not affect ingestion, with an I.I. close to 0 even at the highest tested concentrations (10 mM DEN and 5 mM HAR), but cause a strong aversive choice preference response (Fig. 1*C*). These results indicate that some putative bitter tastants act independently on ingestion and choice preference, two contact chemosensation-driven behavioral paradigms that are commonly viewed as being closely related.

### CAF affects both mouth-hook contraction and bending, while denatonium only affects bending behavior

In measuring taste-driven larval choice preference behavior, the determination of whether a certain chemical is an attractant or a repellent is based on where the larvae are located, i.e., on the control or experimental sectors, after a certain amount of time. We discovered that this method was a source of error in the case of measuring preference behavior to nicotine. While monitoring larvae over the entire 8 min of measurement after the larvae were placed in the center of the plate, we found that most of the larvae stopped movement before the 8 min passed (Extended Data Fig. 1-1). This indicates that larvae are not staying in the nicotine quadrants because they prefer nicotine itself, but because of possible toxic effects of nicotine that block movement. This case of nicotine is an example of misreading actual behavior due to limitations in methodology. After observing this, we decided that additional behavioral assays would be necessary for the accurate analysis of taste-driven larval behavior.

**Figure 2.**
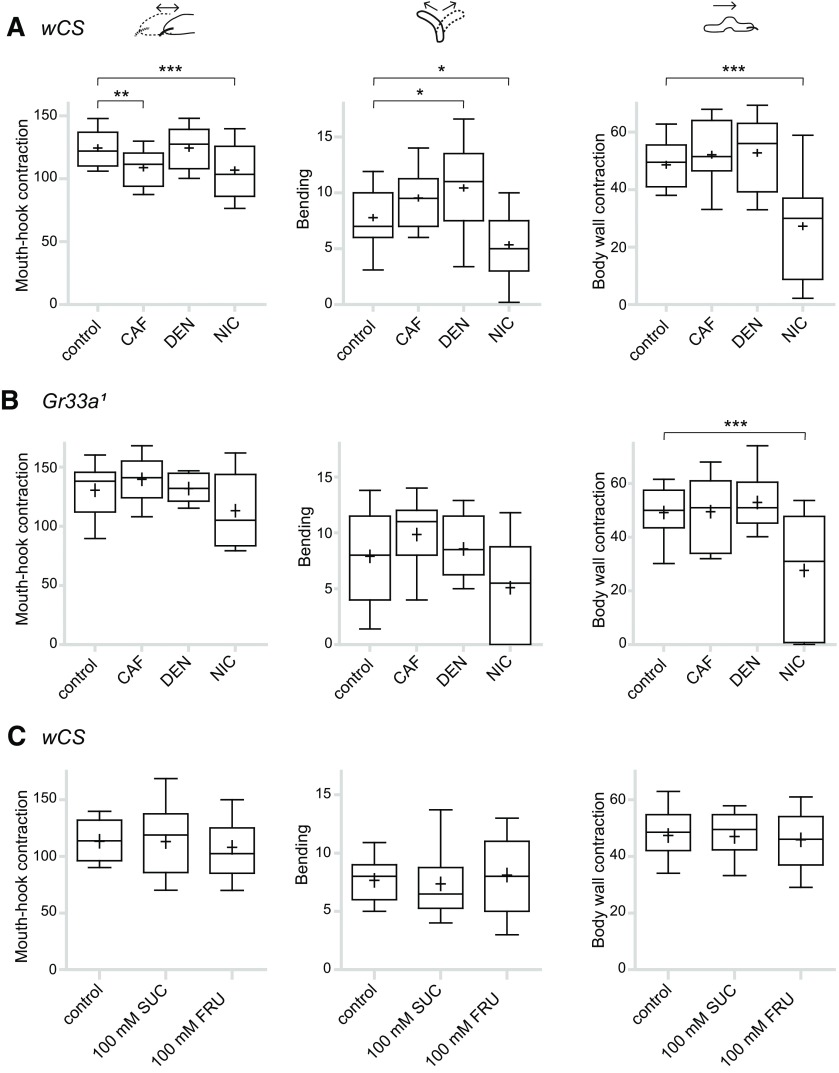
Measurement of larval behavioral responses to given tastants: mouth-hook contractions (left), bending (middle), and body wall contractions (right). Behavioral responses during 1 min for larvae placed on agarose plates containing the indicated tastants, compared with larvae placed on agarose-only control plates. ***A***, Results for *wCS* larvae. For each data point, 16 < *n *<* *40; *p <* *0.05, **p <* *0.01, ***p <* *0.001 versus control, one-way ANOVA followed by Dunnett’s multiple comparison test. ***B***, Results for *Gr33a^1^* larvae. For each data point, 16 < *n *<* *40; ***p <* *0.001 versus control, one-way ANOVA followed by Dunnett’s multiple comparison test. ***C***, Behavioral responses of *wCS* larvae to the indicated sugars and control. For each data point, 16 < *n *<* *40.

Previous studies indicated that the frequency of mouth-hook contractions directly correlates to the amounts ingested (Joshi and Mueller, 1988; Wu et al., 2003, 2005). Also, lateral bending is one of the most commonly observed components of turning behavior, which is evoked when larvae decide to avoid noxious stimuli or explore new territory (Okusawa et al., 2014; Hernandez-Nunez et al., 2015). We were able to correlate these behaviors with larval responses to bitter chemicals. We counted the frequencies of mouth-hook contractions, bending, and body wall contractions on *wCS* larvae exposure to CAF, denatonium, or nicotine (Fig. 2*A*). Mouth-hook contractions were observed to decrease on exposure to CAF compared with the agarose-only control (Fig. 2*A*, left), consistent with the decrease in ingestion (Fig. 1). An increase in bending was observed in response to CAF, although not statistically significant (Fig. 2*A*), and this indicates an increase in turns to avoid an aversive cue. Upon exposure to denatonium, mouth-hook contractions did not show a significant difference from the control (Fig. 2*A*, left), while bending increased (Fig. 2*A*, middle). These behavioral responses were consistent with the behavior tests that showed a neutral response to denatonium in the ingestion assay, and an aversive response in the choice preference test. The frequency of body wall contractions was not affected on exposure to CAF or denatonium (Fig. 2*A*, right), indicating that larval locomotion remained unaffected. However, all indices were observed to decrease on exposure to nicotine, including a strong drop in measured locomotion (Fig. 2*A*). This indicates that nicotine has a toxic or a muscular relaxant effect on larvae, causing an overall inhibition of movement. Thus, these results show that the reason nicotine initially appeared to act as an attractant in choice preference assays (Fig. 1*C*) was due to larvae simply stopping movement after reaching the nicotine-containing quadrants.

In the adult fly, Gr33a was identified as an important co-receptor in bitter-sensitive neurons that act in sensing most non-volatile bitter chemicals, and the *Gr33a* mutant (*Gr33a^1^*) fly is insensitive to most bitter chemicals (Moon et al., 2009). *Gr33a* mutant larvae did not show the changes in mouth-hook contraction and/or bending that were observed in control larvae toward denatonium or CAF (Fig. 2*B*), indicating that these bitter-driven behaviors are mediated by Gr33a-expressing gustatory receptor neurons (GRNs). Body wall contractions of *Gr33a^1^* larvae were observed to drop in the presence of nicotine, similarly to *wCS* larvae (Fig. 2*B*), showing that nicotine indeed inhibits larval movement. This also leads to a subsequent tendency of decrease in mouth-hook contractions and bending (Fig. 2*B*). In contrast to bitter chemicals, the measured phenotypes did not change noticeably on exposure to sugar (Fig. 2*C*), indicating that these behaviors are not useful parameters to measure feeding enhancement to attractive cues, likely because larvae feed by default.

### DP1 neurons are necessary and sufficient for CAF-induced ingestion reduction and avoidance

Although both CAF and denatonium induce aversive behavior, CAF decreases both preference and ingestion, while denatonium has a strong inhibitory effect on only preference. This suggests that independent circuits may exist to detect different bitter tastants and to mediate distinct behavioral steps in feeding. To examine this possibility and identify the initial sensory neurons of the neural circuits that mediate choice preference or ingestion, we aimed to identify differences in the cells that detect CAF and denatonium. For this purpose, we first used specific *Gr-GAL4* drivers and the inward-rectifier potassium channel *UAS-Kir2.1* to inhibit the activity of specific larval gustatory neurons.

**Figure 3.**
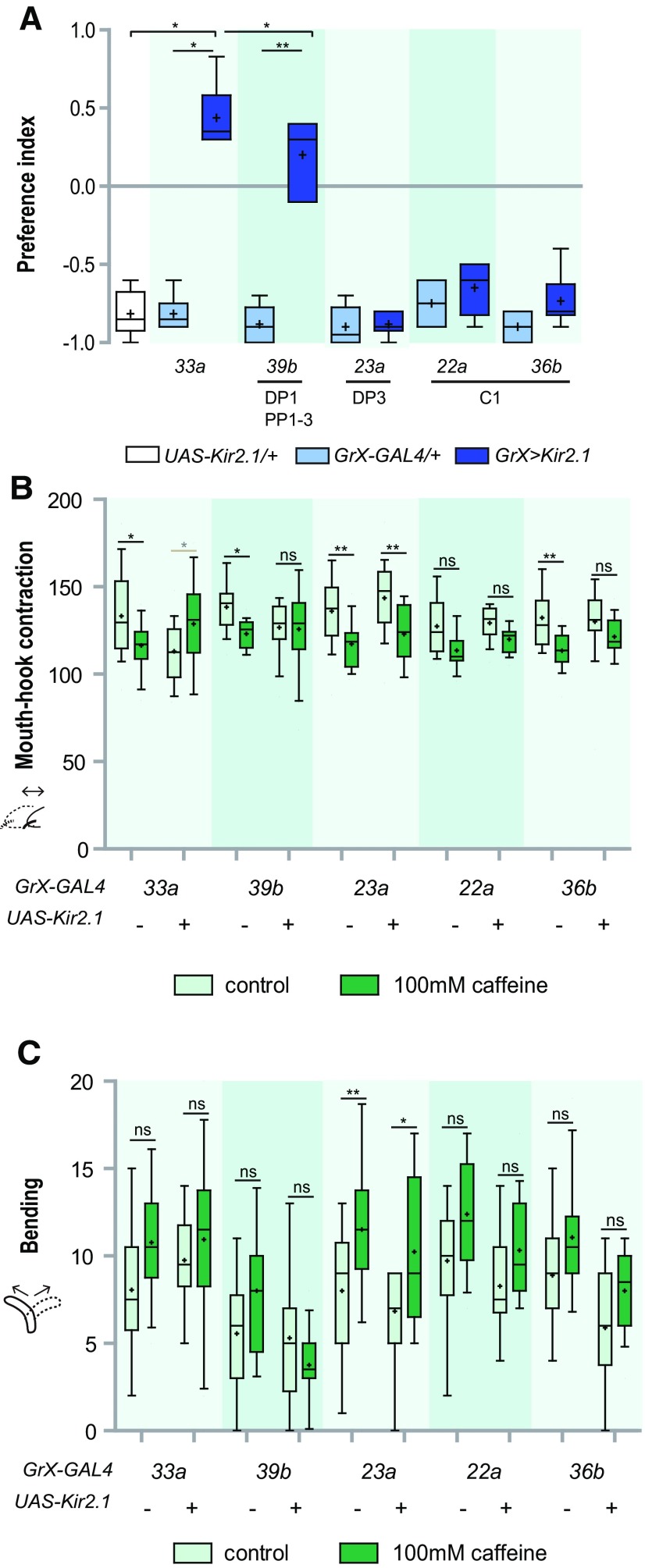
A subset of pharyngeal neurons including DP1 mediates CAF-induced avoidance and changes in mouth-hook contraction and bending behaviors. ***A***, Comparison of preference behavior in response to 100 mM CAF when *Gr-GAL4* drivers specifically expressed in the GRNs listed under the underlines were used to block neuronal activity. For each data point, *n *=* *6. *p <* *0.05, **p <* *0.01, Kruskal–Wallis test followed by Dunn’s multiple comparison test. ***B***, Comparison of reduction of mouth-hook contractions in response to 100 mM CAF when *Gr-GAL4* drivers specifically expressed in the TO or pharyngeal organs were used to block GRN activity. For each data point, 17 < *n *<* *30; *p <* *0.05, **p <* *0.01, two-way ANOVA followed by the Bonferroni *post hoc* test. *Gr33a*>*Kir2.1* larvae in response to 100 mM CAF was marked in a gray asterisk to distinguish it from other data, since mouth-hook contractions were increased compared with other data. ns, not significant. ***C***, Comparison of increase of bending in response to 100 mM CAF when *Gr-GAL4* drivers specifically expressed in the TO or pharyngeal organs were used to block GRN activity. For each data point, 17 < *n *<* *30; *p <* *0.05, **p < 0.01 versus control, two-way ANOVA followed by the Bonferroni *post hoc* test. ns, not significant. + and – indicate whether the transgenes are present or absent.

In a previous study, we found that a specific pair of neurons in the DPS called DP1 is necessary and sufficient for CAF-mediated decrease in ingestion (Choi et al., 2016). An independent study found that the same neurons mediate CAF avoidance (Apostolopoulou et al., 2016). The DP1 neuron expresses the *Gr33a*-, *Gr39b-*, and *Gr22d-GAL4* drivers (Choi et al., 2016). Consistent with our previous study, CAF induces avoidance in the present choice preference assay, and this aversive effect is reversed when the activity of *Gr33a*- or *Gr39b-GAL4*-expressing neurons is inhibited, but not when inhibiting DP3 or C1 neurons (Fig. 3*A*). Also, CAF-induced decrease of mouth-hook contractions was reversed on inhibition of *Gr33a*- or *Gr39b-GAL4*-expressing neuron activity (Fig. 3*B*). In contrast, CAF-induced decrease of mouth-hook contractions was unaffected on inhibition of the DP3 neuron (Fig. 3*B*). A similar trend was observed on inhibition of the C1 neuron, albeit the changes were not statistically significant (Fig. 3*B*). Although the variation in CAF-induced increase in bending was generally too large to come to a statistically significant conclusion, a general tendency of reversal was observed on inhibition of *Gr39b-GAL4*-expressing neuron (DP1) activity, but not on inhibition of DP3 or C1 neurons (Fig. 3*C*). Thus, DP1 is the main mediator of CAF-induced avoidance and changes in ingestion, mouth-hook contraction, and bending behaviors.

**Figure 4.**
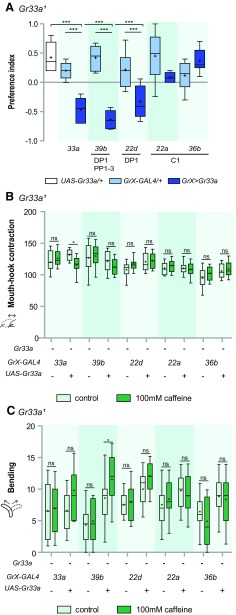
DP1 is sufficient for CAF-induced aversive responses in ingestion and choice preference. ***A***, Choice preference in response to 100 mM CAF on expression of Gr33a in the *Gr33a* mutant using the indicated *Gr-GAL4* drivers for specific expression in the GRNs listed under the underlines. For each data point, 6 < *n *<* *12; ***p < 0.001 versus *GAL4* and *UAS* control, one-way ANOVA followed by the Newman–Keuls method. ***B***, Comparison of reduction of mouth-hook contractions in response to 100 mM CAF on expression of Gr33a in the *Gr33a* mutant using the indicated *Gr-GAL4* drivers for specific expression in the GRNs. For each data point, 18 < *n* < 28; *p <* *0.05, two-way ANOVA followed by the Bonferroni *post hoc* test. ns, not significant. ***C***, Comparison of increase in bending in response to 100 mM CAF on expression of Gr33a in the *Gr33a* mutant using the indicated *Gr-GAL4* drivers for specific expression in the GRNs. For each data point, 18 < *n *<* *28; *p <* *0.05, two-way ANOVA followed by the Bonferroni *post hoc* test. ns, not significant. + and – indicate whether transgenes are present or absent.

Next, we attempted to rescue behavioral defects in *Gr33a^1^* larvae that do not show CAF-sensitive behaviors, by expressing a wild-type *Gr33a* transgene in specific GRNs. *Gr33a^1^* behavior in choice preference was rescued on expression of Gr33a in *Gr33a*-, *Gr39b-*, or *Gr22d-GAL4*-expressing neurons (Fig. 4*A*). DP1 is commonly expressed by all three of these *Gr-GAL4* drivers. In the case of mouth-hook contractions, *Gr33a^1^* defects were only rescued when Gr33a was expressed in *Gr33a*-*GAL4*-expressing neurons, although when Gr33a was expressed in only DP1, mouth-hook contractions tended to decrease on exposure to CAF compared with the *GAL4*-only control (Fig. 4*B*). Also, in the case of bending, *Gr33a^1^* defects were only rescued when Gr33a was expressed in *Gr39b*-*GAL4*-expressing neurons, while bending showed a tendency to increase when Gr33a was expressed in DP1 and not when Gr33a was expressed in the C1 neurons (Fig. 4*C*). Thus, Gr33a function in DP1 neurons is sufficient for all types of CAF-driven larval aversive behavior tested.

### Gustatory neurons in the TO mediate denatonium-induced avoidance behavior

Our behavioral experiments indicated that denatonium did not affect ingestion and only affected choice preference. The *Drosophila* larval C7 neuron is known to be necessary and sufficient for denatonium sensing (van Giesen et al., 2016b). Also, Gr59c is known to function in denatonium sensing in the adult fly labellum (Weiss et al., 2011; Freeman et al., 2014; Sung et al., 2017), and *Gr59c-GAL4* is specifically expressed in only one pair of larval GRNs, the C1 neuron pair, which houses expression of the highest number of *Gr-GAL4* drivers in the TO, the major external gustatory organ of *Drosophila* larva (Kwon et al., 2011). Therefore, we tested the roles of the TO C1 and C7 neurons in denatonium sensing.

**Figure 5.**
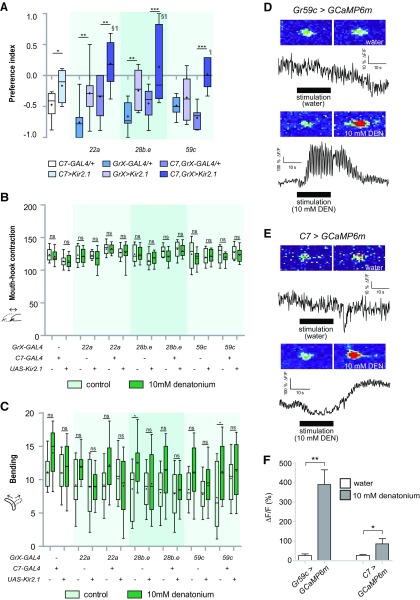
Two pairs of GRNs in the TO, C1 and C7, detect denatonium to induce avoidance. ***A***, Comparison of preference behavior in response to 10 mm denatonium when *GAL4* drivers specifically expressed in C1 or C7, GRNs in the TO, were used to block neuronal activity. For each data point, 6 < *n *<* *10; *p <* *0.05, **p <* *0.01, ***p <* *0.001, one-way ANOVA followed by uncorrected Fisher’s LSD test. Symbols § and ¶ each represent the significance versus *C7>Kir2.1* and *GrX>Kir2.1*. ***B***, Comparison of reduction of mouth-hook contractions in response to 10 mM denatonium when *GAL4* drivers specifically expressed in the TO were used to block GRN activity in C1 and/or C7. For each data point, 18 < *n *<* *20. Two-way ANOVA followed by the Bonferroni *post hoc* test. ns, not significant. ***C***, Comparison of increase of bending in response to 10 mm denatonium when *GAL4* drivers specifically expressed in the TO were used to block GRN activity in C1 and/or C7. For each data point, 18 < *n *<* *20; *p <* *0.05, two-way ANOVA followed by the Bonferroni *post hoc* test. ns, not significant. “+” and “-” indicate whether transgenes are present or absent. ***D***, ***E***, Calcium currents can be measured in the C1 (***D***) and C7 (***E***) neurons before and during the application of 10 mM denatonium using the genetically encoded calcium sensor GCaMP6m. ***F***, TO C1 and C7 neurons, labeled by *Gr59c-GAL4* and *C7-GAL4*, respectively, showed neuronal activity to 10 mM denatonium. For each data point, 6 < *n *<* *30; *p <* *0.05, **p <* *0.01, Mann–Whitney *U* test pair-wise comparisons of water control and 10 mM denatonium.

We used 3 *Gr-GAL4* drivers (*Gr22a*-, *Gr28b.e*-, and *Gr59c-GAL4*) that are only expressed in C1 and *C7-GAL4* to inhibit the activity of either C1 or C7, or both C1 and C7 simultaneously, and examined larval preference to denatonium. Avoidance of denatonium decreased on inhibition of either C1 or C7, and an additive decrease was observed on inhibition of both C1 and C7 (Fig. 5*A*). Consistent with our observations that denatonium did not affect mouth-hook contractions (Fig. 2), inhibition of C1 and C7 during denatonium exposure did not affect the mouth-hook contractions of larvae (Fig. 5*B*). In contrast, inhibition of either C1 or C7 resulted in a tendency to decrease denatonium-induced increase of larval bending behavior (Fig. 5*C*). These results suggest that C1 and C7 may work together to sense denatonium and induce avoidance behavior in *wCS* larvae. Consistently, intracellular calcium concentration was increased in these neurons during stimulation with denatonium (Fig. 5*D–F*).

### Activation of DP1 is sufficient to cause the suppression of ingestion

**Figure 6.**
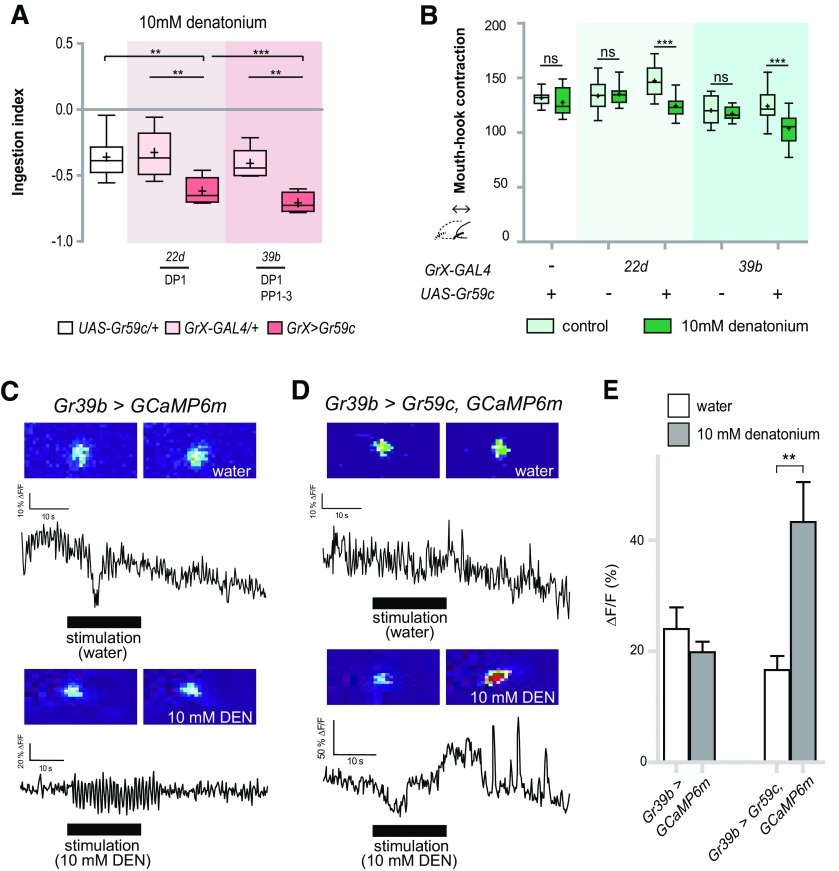
Detection of denatonium by DP1, a pair of dorsal pharyngeal neurons, causes suppression of ingestion. ***A***, Comparison of ingestion in response to 10 mm denatonium when *Gr22d-GAL4* and *Gr39b-GAL4* drivers were used to ectopically express Gr59c in DP1. For each data point, 6 < *n *<* *14; **p <* *0.01, ***p <* *0.001 versus *GAL4* and *UAS* control, one-way ANOVA followed by the Newman–Keuls method. ***B***, Comparison of reduction of mouth-hook contractions in response to 10 mM denatonium when *Gr22d-GAL4* and *Gr39b-GAL4* drivers were used to ectopically express Gr59c in DP1. For each data point, 18 < *n *<* *20; ***p <* *0.001, two-way ANOVA followed by the Bonferroni *post hoc* test. ns, not significant. + and – indicate whether transgenes are present or absent. ***C***, ***D***, Calcium currents were measured in DP1 neurons with *Gr39b-GAL4* used to express GCaMP6m only (***C***) or GCaMP6m and Gr59c together (***D***) before and during the application of 10 mm denatonium. ***E***, Changes in neuronal activity in response to 10 mM denatonium was measured in the DP1 neuron with ectopically expressed Gr59c. For each data point, 6 < *n *<* *11; **p <* *0.01, Mann–Whitney *U* test pair-wise comparisons of water control and 10 mM denatonium.

We tested whether activation of the DP1 neuron by denatonium could induce a change in ingestion, which is not normally affected by denatonium. For this purpose, we used Gr59c, which is known to be critical for denatonium sensing in the adult fly (Weiss et al., 2011; Freeman et al., 2014; Sung et al., 2017). Based on GAL4 reporter expression, Gr59c is not expressed in larval pharyngeal neurons (Kwon et al., 2011). We expressed Gr59c in DP1 using two *Gr-GAL4* drivers, *Gr22d*- and *Gr39b-GAL4*, and examined the ingestion of denatonium. Larvae were observed to show a decrease in ingestion compared with the control (Fig. 6*A*), as well as a decrease in mouth-hook contractions (Fig. 6*B*). In addition, DP1 neurons ectopically expressing Gr59c were observed to show an increase in cellular calcium levels on exposure to denatonium (Fig. 6*C–E*). These results indicate that activation of the pharyngeal GRN DP1 by a specific substrate is sufficient to suppress the ingestion of that substrate. Thus, *wCS* larvae exposed to denatonium show a change only in choice preference but not ingestion, because denatonium is only detected by the TO and not by the pharyngeal organs.

## Discussion

A general assumption would be that a tastant would cause a similar response in ingestion and choice preference behavior, in either a positive or negative manner. However, our findings corroborate that certain tastants elicit divergent ingestion and choice preference behavior. Combining molecular genetic tools, behavioral assays, and genetically encoded calcium sensors to assess neuronal activity, our results provide evidence that relatively independent neural systems appear to regulate the two initial processes of feeding in *Drosophila* larva: searching for palatable food, i.e., choice preference, and eating the selected food, i.e., ingestion. We show that a subset of gustatory neurons housed in the TO, the external gustatory organ of *Drosophila* larva, detect denatonium and induce avoidance behavior, and DP1, a specific pair of GRNs in the dorsal pharyngeal organ, plays a major role in regulating both ingestion and avoidance in response to CAF.

The TO of *Drosophila* larva is located at the tip of the cephalic lobes, and is thus anatomically likely to be the first organ to contact external stimuli and subsequently cause a change in movement to regulate the initial step of feeding. Similarly, pharyngeal sensilla are located between the external sense organs and digestive organs, and are thus anatomically likely to act in maintaining the ingestion of appetitive foods while stopping ingestion and causing avoidance of aversive cues such as bitter toxins. It could be advantageous for ingestion to be predominantly controlled by pharyngeal sense organs, rather than by external organs, since animals can try out a potential food source before making their decision, rather than blindly avoiding it. This could be a particularly advantageous strategy for insect larvae whose main purpose is to feed. Also, the difference in behavioral responses elicited by the C1 and C7 neurons in the TO and DP1 in the pharyngeal sense organs is likely linked to the difference in brain projection patterns of GRNs from the TO and pharyngeal GRNs from the larval SEZ (Kwon et al., 2011), with the distinct projection areas of the brain taste center likely being linked to different circuits, resulting in distinct behavioral outputs.

In *Drosophila* larvae, choice and ingestion have generally been grouped together and studied as a group of reflexive behaviors (Schipanski et al., 2008; El-Keredy et al., 2012; Rohwedder et al., 2012; Apostolopoulou et al., 2014). Sugar processing provides another intriguing example of divergence between choice and ingestion. Larvae generally show increased preference and feeding when exposed to increasing concentrations of fructose or sucrose. At extremely high concentrations such as 2 M or 4 M, larvae still exhibit preference in terms of choice, but show suppression of feeding (or ingestion, as we denote it here; Schipanski et al., 2008; Rohwedder et al., 2012). Since this suppression of ingestion could be due to high viscosity and/or osmolarity, a direct comparison to the processing of aversive tastants such as bitter chemicals is difficult. However, this example nonetheless provides evidence that relatively independent circuits exist to determine choice and ingestion. Using bitter tastants, we find that choice and ingestion can manifest in clearly divergent behaviors to the same compounds and elucidate the cellular basis of these observations. Similarities to the observation that external sense organs and pharyngeal organs appear to be involved in somewhat independent behavioral output can be seen in sugar consumption in the adult fly. The activation of sweet GRNs in the legs and labellum initiates feeding behaviors including the proboscis extension reflex, and pharyngeal sweet GRNs play an important role in directing the sustained consumption of sweet compounds (LeDue et al., 2015).

Most of the 22 putative bitter tastants tested here, including CAF, cause negative effects in choice preference and ingestion. Nicotine caused a positive P.I. in the choice preference assay. Although we cannot completely rule out the possibility that nicotine could act as an attractive chemosensory cue at low concentrations, we found that nicotine inhibits the movement of larvae in our experimental setup. Larvae strongly avoid denatonium, but once they sample denatonium-containing food, they ingest it. This ingestion likely occurs because denatonium is added to the agarose of the entire plate, whereas larvae probably would not ingest as much if they had the choice. Nevertheless, our results suggest that this larval response to denatonium is due to the existence of a functional receptor complex for denatonium in the TO, which does not exist in the pharyngeal sense organs, or at the very least the DP1 neuron. Consistently, ectopic expression of GR59c in DP1 caused a novel calcium response to denatonium and suppression of ingestion in response to denatonium. Some remaining questions regarding sensing of denatonium merit further study. Avoidance to denatonium is defective when either C1 or C7 is inactivated, indicating that C1 and C7 are not redundant in terms of behavior. It is possible that a certain threshold of neuronal activity is required to elicit behavior, or inactivation of one neuron may cause a change in the functions of other GRNs. Although a numerically simple system, larval GRNs also have a multimodal character (van Giesen et al., 2016b), and as such a more complicated mechanism might be involved. Also, in the bitter sensing neurons of the adult labellum, two complexes, GR32a/GR66a/GR59c and GR32a/GR66a/GR22e, are each sufficient to confer a response to denatonium (Sung et al., 2017). Based on *Gr-GAL4* expression, the larval DP1 neuron expresses Gr22e, but not Gr59c (Choi et al., 2016), but is not capable of detecting denatonium. This suggests that the GRNs of the larva and adult fly possess different cellular contexts, which could be interesting to unravel. An interesting remaining question is if Gr59c is solely responsible for denatonium sensing in the larval C1 neuron or if the existing Gr22e can rescue denatonium sensing in *Gr59c* mutants. This would indicate that Gr22e needs a specific co-receptor repertoire for denatonium detection and could help elucidate coding differences in the larva versus the adult fly.

The levels at which distinct bitter compounds are detected might reflect the ecological niche of the animal and the toxicity level of a given tastant. Our results suggest that information from the DP1 neuron is processed in a circuit that results in negative and aversive behavior in ingestion and choice preference to CAF. The C1 and C7 neuron in the TO elicit avoidance to denatonium in choice preference behavior. Thus, these results suggest that distinct sensory neurons appear to have distinct sensory roles, likely through the expression of specific receptors or specific groups of receptors. Sensory information detected by these sensory neurons appears to be processed through distinct circuits in the central nervous system to mediate changes in ingestion or choice behavior. It is yet unclear whether the different circuits interact to result in a final behavioral output. Further examination of the potential connections between the external and pharyngeal gustatory neurons and interneurons or motor neurons in the brain may provide insight into the overall neural circuit that regulates feeding and locomotion.

## Synthesis

Reviewing Editor: Christian Lüscher, University of Geneva Decisions are customarily a result of the Reviewing Editor and the peer reviewers coming together and discussing their recommendations until a consensus is reached. When revisions are invited, a fact-based synthesis statement explaining their decision and outlining what is needed to prepare a revision will be listed below. The following reviewer(s) agreed to reveal their identity: NONE. Note: If this manuscript was transferred from JNeurosci and a decision was made to accept the manuscript without peer review, a brief statement to this effect will instead be what is listed below.

After consultation with the referees there are a number of statistical issues that we would like to see addressed before making a final decision:

1) Fig 3B, 3C, 4B, 4C, 5B, 5C, 6B should all be done with 2-way ANOVAs and interactions between genotype and treatment should be tested to see whether the genotype has an effect on the response to bitter.

2) In Figure 5A, the control data should not be used three separate times.

3) Figure 2 should be a Dunnett’s test because they are always comparing to a single control.

4) Figure 1 should have statistics.

## Author Response

We are pleased to submit our revised manuscript (“Cellular basis of bitter-driven aversive behaviors in Drosophila larva” by Choi et al.) to eNeuro. As you will see, we have addressed all of the statistical issues raised by the referees. Please see our responses to each of their comments below:

1) Fig 3B, 3C, 4B, 4C, 5B, 5C, 6B should all be done with 2-way ANOVAs and interactions between genotype and treatment should be tested to see whether the genotype has an effect on the response to bitter.

-- For Figures 3B, 3C, 4B, 4C, 5B, 5C, and 6B, two-way ANOVA followed by the Bonferroni post-test was performed. We accordingly made changes to the Results, “Experimental design and statistical analyses” section of the Materials and Methods, as well as each figure legend. The interpretation of the experimental results basically remains the same, but the wording was changed from “showed a statistically significantly different result” to “showed a tendency to differ.” The edited parts of the Results section are as follows: Line 292, “An increase in bending was observed in response to caffeine, although not statistically significant (Fig. 2A), and this indicates an increase in turns to avoid an aversive cue.”; Line 313, “ Body wall contractions of Gr33a1 larvae were observed to drop in the presence of nicotine, similarly to wCS larvae (Fig. 2B), showing that nicotine indeed inhibits larval movement. This also leads to a subsequent tendency of decrease in mouth-hook contractions and bending (Fig. 2B).”; Line 343, “In contrast, caffeine-induced decrease of mouth-hook contractions was unaffected upon inhibition of the DP3 neuron (Fig. 3B). A similar trend was observed upon inhibition of the C1 neuron, albeit the changes were not statistically significant (Fig. 3B). Although the variation in caffeine-induced increase in bending was generally too large to come to a statistically significant conclusion, a general tendency of reversal was observed upon inhibition of Gr39b-GAL4-expressing neuron (DP1) activity, but not upon inhibition of DP3 or C1 neurons (Fig. 3C). Thus, DP1 is the main mediator of caffeine-induced avoidance and changes in ingestion, mouth-hook contraction, and bending behaviors.”; Line 364, “Also in the case of bending, Gr33a1 defects were only rescued when Gr33a was expressed in Gr39b-GAL4 expressing neurons, while bending showed a tendency to increase when Gr33a was expressed in DP1 and not when Gr33a was expressed in the C1 neurons (Fig. 4C).”; Line 387, “In contrast, inhibition of either C1 or C7 resulted in a tendency to decrease denatonium-induced increase of larval bending behavior (Fig. 5C).”

2) In Figure 5A, the control data should not be used three separate times.

-- We corrected the figure so the control data is only shown once.

3) Figure 2 should be a Dunnett’s test because they are always comparing to a single control.

-- We corrected the figure as the reviewer pointed out. For Figure 2, one-way ANOVA followed by Dunnett’s multiple comparison test was performed.

4) Figure 1 should have statistics.

-- Figure 1 provides a description of preference and ingestion behavior to various tastants, and is not a comparison of the behaviors. Thus the figure does not need statistics.
